# Proline Isomerization as a Key Determinant for Hsp90-Toxin Interactions

**DOI:** 10.3389/fcimb.2021.771653

**Published:** 2021-10-22

**Authors:** Alisha Kellner, Patrick Cherubin, James K. Harper, Ken Teter

**Affiliations:** ^1^ Burnett School of Biomedical Sciences, University of Central Florida, Orlando, FL, United States; ^2^ Department of Chemistry and Biochemistry, Brigham Young University, Provo, UT, United States

**Keywords:** AB toxins, chaperone, cholera toxin, Hsp90, peptidyl prolyl *cis-trans* isomerase, toxin translocation

## Abstract

The A chains of ADP-ribosylating toxins exploit Hsp90 for translocation into the host cytosol. Here, we hypothesize that *cis* proline residues play a key role in toxin recognition by Hsp90. Our model is largely derived from studies on the unusual interplay between Hsp90 and the catalytic A1 subunit of cholera toxin (CTA1), including the recent identification of an RPPDEI-like binding motif for Hsp90 in CTA1 and several other bacterial toxins. *Cis/trans* proline isomerization is known to influence protein-protein interactions and protein structure/function, but it has not yet been proposed to affect Hsp90-toxin interactions. Our model thus provides a new framework to understand the molecular basis for Hsp90 chaperone function and Hsp90-driven toxin translocation.

## Introduction

AB toxins contain an enzymatically active A subunit and a cell-binding B subunit (Zuverink and Barbieri, 2018). They are secreted into the external environment but attack targets within the cytosol of a host cell. To reach their cytosolic targets, AB toxins bind to the cell surface and are internalized by receptor-mediated endocytosis. Some toxins respond to the acidified lumen of the endosomes with a structural change in the B subunit that forms a channel in the endosome membrane. The A subunit then utilizes the toxin-generated pore to access the cytosol (Barth et al., 2004). Other AB toxins travel to the endoplasmic reticulum (ER) before toxin disassembly and A chain passage into the cytosol through a host “translocon” pore in the ER membrane (Teter, 2013).

Many AB toxins contain catalytic subunits that modify their cytosolic targets through ADP-ribosylation (Simon et al., 2014). The Barth lab has proposed that all of these ADP-ribosylating toxins (ADPRTs) require an interaction with the host chaperone Hsp90 for translocation to the cytosol (Barth, 2011; Ernst et al., 2017). There is considerable experimental evidence for this model, but the structural basis for Hsp90 interaction remains unknown. Here, we hypothesize that Hsp90 specifically recognizes the *cis* isomer of proline in the toxin A chain.

### Proline Isomerization

Most Xaa-Xaa peptide bonds (with Xaa representing any amino acid) are in the *trans* state but can, under certain conditions, rotate 180° to the *cis* conformation. Proline is the most common amino acid for *cis*/*trans* isomerization because the *cis* and *trans* forms are almost isoenergetic, so neither form is strongly favored. Proline isomerization plays a critical role in protein folding and several cellular processes, as the *cis* and *trans* forms of a Xaa-Pro bond can each independently influence protein structure/function and protein-protein interactions (Andreotti, 2003; Lu et al., 2007; Joseph et al., 2012). For example, protein disulfide isomerase specifically recognizes the *cis* configuration of two Xaa-Pro bonds in RNase T1. Its interaction with RNase T1 is greatly enhanced by the *trans*-to-*cis* isomerization of these two bonds by cyclophilin A, a peptidyl prolyl *cis*-*trans* isomerase (PPI) (Schönbrunner and Schmid, 1992). We predict a similar interaction between Hsp90 and the *cis* conformation of proline in the A chains of ADPRTs.

### Hsp90, A Molecular Chaperone

Hsp90 exhibits a greater level of substrate selectivity than other chaperones, yet it is not thought to recognize any specific sequence or structural motif. Instead, it usually partners with co-chaperones for substrate binding (Pearl and Prodromou, 2006; Li et al., 2012; Karagoz and Rudiger, 2015; Schopf et al., 2017). Processing often begins with the binding of another chaperone, Hsp40. This facilitates the sequential recruitment of Hsp70, Hsp70-Hsp90 organizing protein (Hop), and, finally, Hsp90. Other co-chaperones such as PPIs may further regulate the interaction of Hsp90 with its client protein. ATP hydrolysis by substrate-bound Hsp90 converts the chaperone to an open conformation, releases the processed client protein, and resets the chaperone cycle. This general process - including the involvement of co-chaperones - is frequently linked to the translocation and/or cytosolic refolding of toxin A chains.

### Toxin Translocation From the Endosomes to the Cytosol

After internalization into the host cell, the A chains of endosome-translocating toxins respond to the acidified vesicle lumen by shifting to an unfolded or partially unfolded state. Some toxins then use a pH-driven ratchet mechanism for A chain delivery to the cytosol (Krantz et al., 2006). For other toxins, the A chain is extracted from the endosomes by chaperones and other proteins in the host cytosol. The subset of endosome-translocating ADPRTs exploits Hsp90 for this purpose (Haug et al., 2003; Ratts et al., 2003; Haug et al., 2004; Kaiser et al., 2011; Lang et al., 2014; Schuster et al., 2017). These toxins also require host PPI activity for A chain passage into the cytosol (Kaiser et al., 2009; Kaiser et al., 2011; Kaiser et al., 2012; Barth, 2013; Lang et al., 2014; Ernst et al., 2015; Schuster et al., 2017). Hsp90 and PPIs may interact with a common structural feature of ADPRTs, but a specific chaperone binding site has only been identified for Hsp90 recognition of ER-translocating ADPRTs (Kellner et al., 2019).

### Toxin Translocation From the ER to the Cytosol

The A chains of ER-translocating toxins shift to a disordered state upon holotoxin disassembly (Teter, 2013). Unfolding results from an interaction with the anionic phospholipids of the ER membrane or the intrinsic instability of the free A chain (Day et al., 2002; Pande et al., 2006; Pande et al., 2007; Mayerhofer et al., 2009; Kim et al., 2011). In either case, A chain unfolding activates the ER-associated degradation (ERAD) mechanism for export to the cytosol. Refolding of the cytosolic toxin, along with a paucity of lysine residues required for ubiquitin conjugation, allows the A chain to evade the ubiquitin-dependent proteasomal degradation that usually accompanies ERAD-mediated export to the cytosol (Hazes and Read, 1997; Deeks et al., 2002; Rodighiero et al., 2002; Worthington and Carbonetti, 2007).

Cholera toxin (CT) and several other ER-translocating toxins require Hsp90 for productive intoxication. For CT, Hsp90 binds to an N-terminal RPPDEI sequence in the catalytic CTA1 subunit and then couples CTA1 refolding with CTA1 extraction from the ER: refolding of the toxin as it emerges at the cytosolic face of the ER membrane prevents the folded toxin from sliding back into the narrow diameter of the translocon pore, thus ensuring unidirectional movement out of the ER (Taylor et al., 2010; Burress et al., 2014; Kellner et al., 2019). Mutagenesis studies have demonstrated both RPPDEI prolines are required for CTA1 export to the cytosol (Kellner et al., 2019). Continued association of Hsp90 with the cytosolic pool of CTA1 maintains the toxin in a folded, active conformation for the ADP-ribosylation of its G protein target (Banerjee et al., 2014; Burress et al., 2014).

**Figure 1:**
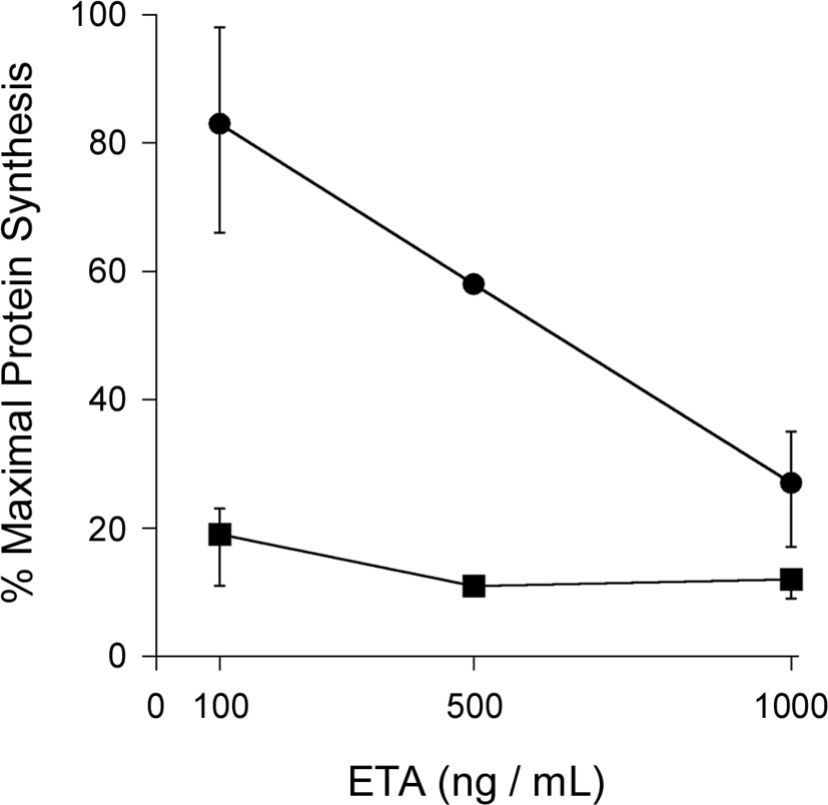
PPI inhibition does not protect cultured cells from ETA. Untreated CHO cells (circles) and CHO cells treated with 10 μM of the cyclophilin inhibitor CsA (squares) were incubated with various concentrations of ETA for 4 h before the extent of protein synthesis was recorded. Maximal protein synthesis was established from parallel samples of unintoxicated cells. Data are presented as the averages ± ranges of 2 (500 ng/mL) or 4 (100, 1000 ng/mL) independent experiments.

**Figure 2:**
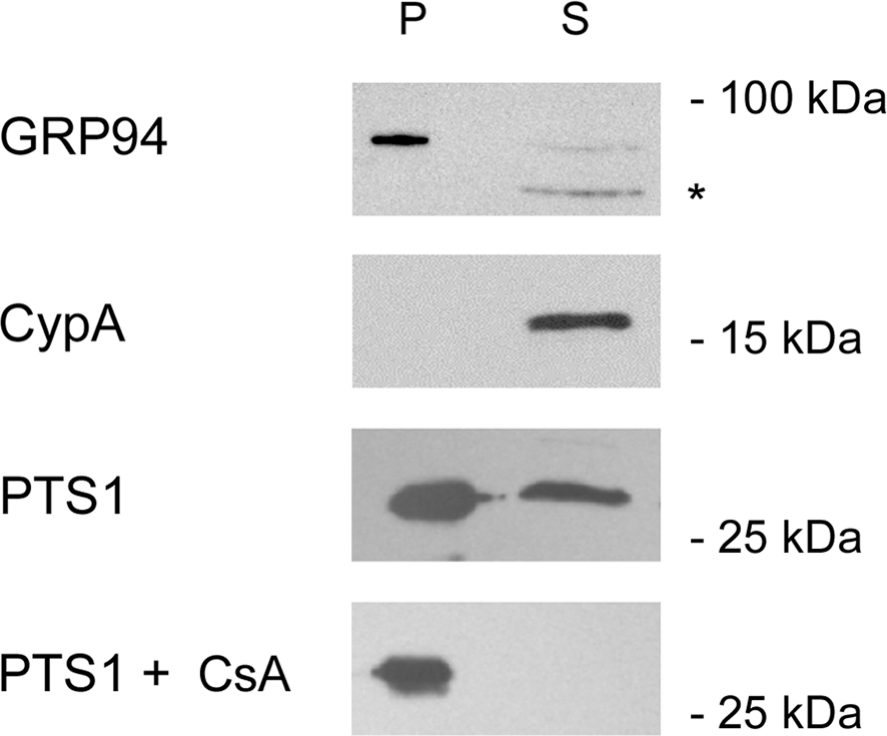
Cyclophilin inhibition blocks PTS1 translocation to the cytosol. CHO cells were mock transfected or transfected with an expression plasmid that directs PTS1 for co-translational insertion into the ER. The toxin is then dislocated from the ER into the cytosol. At 24 h post-transfection, permeabilization of the plasma membrane with digitonin was used in conjunction with differential centrifugation to separate cell extracts into two fractions: a pellet (P) containing intact organelles and a supernatant (S) containing the cytosol. Both fractions from mock-transfected cells were probed by Western blot with antibodies against the lumenal ER protein GRP94 or the cytosolic protein cyclophilin A (CypA). Fractions from PTS1-expressing cells incubated in the absence or presence of cyclophilin inhibitor CsA were probed by Western blot with an antibody against PTS1. The asterisk denotes a non-specific band, most likely due to the secondary antibody, also detected in the cropped region of the CypA and PTS1 blots. One of three representative results is shown.

**Figure 3:**
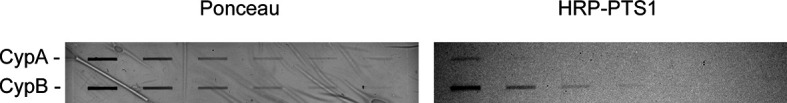
Cyclophilin binding to PTS1. Two-fold serial dilutions of cyclophilin A (CypA) or cyclophilin B (CypB) were placed on nitrocellulose with a slot blot apparatus. Protein loading was confirmed by Ponceau stain (left panel). The membrane was then incubated overnight at 4°C in the presence of HRP-labeled PTS1. Chemiluminescence visualized the bound toxin (right panel). One of two representative experiments is shown. The previously established association between PTS1 and cyclophilin A (Ernst et al., 2018; Ernst et al., 2021) was barely detected in this assay, possibly because our assay used an HRP-conjugated PTS1 subunit whereas previous dot blot assays used a signal amplification strategy with primary and HRP-labeled secondary antibodies to detect the bound PTS1. Our results with HRP-conjugated PTS1 suggest the association of PTS1 with cyclophilin B is stronger than the binding of PTS1 to cyclophilin A.

Other ER-translocating ADPRTs also contain an N- or C-terminal RPPDEI-like motif in the toxin A chain that is recognized by Hsp90 (Kellner et al., 2019). Several of these toxins require Hsp90 for productive intoxication, whereas the A chains from ER-translocating toxins that are not ADPRTs do not contain a RPPDEI motif and do not utilize Hsp90 for export to the cytosol (**Table 1**). Endosome-translocating toxins also lack the RPPDEI motif. These observations suggest ER-translocating ADPRTs exploit a common Hsp90-dependent mechanism that is distinct from the Hsp90 pathway followed by endosome-translocating ADPRTs. Additional support for this possibility is derived from the role of PPIs in toxin translocation: this chaperone family is essential for intoxication with endosome-translocating ADPRTs (Barth, 2013) but is not necessary for CT activity against cultured cells (Burress et al., 2019). Like CT, *Pseudomonas aeruginosa* exotoxin A (ETA) can affect cells treated with the PPI inhibitor cyclosporin A (CsA). In fact, CsA actually sensitizes cells to both ETA (**Figure 1**) and ETA-based immunotoxins (Andersson et al., 2009). It should be noted that another ER-translocating ADPRT - pertussis toxin (PT) - does require a PPI for intoxication. CsA thus blocks the ER-to-cytosol translocation of the catalytic PTS1 subunit (**Figure 2**) (Ernst et al., 2018) and resulting intoxication of cultured cells (Ernst et al., 2018). However, CsA also inhibits the ER-localized PPI cyclophilin B (Bernasconi et al., 2010) and may therefore disrupt host interactions with PTS1 before its extraction to the cytosol. Preliminary experiments have documented the binding of cyclophilin B to PTS1, and the affinity between cyclophilin B and PTS1 is apparently higher than the established association between cyclophilin A and PTS1 (**Figure 3**). The functional role of cyclophilin B binding to PTS1 remains to be established.

**Table 1:** An RPPDEI-like motif at the termini of ER-translocating ADPRTs.

	A chain RPPDEI-like motif	Hsp90 binding to toxin terminus	Hsp90 role in intoxication	Hsp90 role in translocation	References
ADPRTs					
CT	N-terminal **RPPDEI**	+	+	+	(Taylor et al., 2010; Burress et al., 2014; Kellner et al., 2019)
PT	N-terminal **RPPEDV**	+	+	+	(Kellner et al., 2019; Ernst et al., 2021)
ETA	C-terminal **PGKPPR**	+	+	nd	(Taylor et al., 2015; Kellner et al., 2019)
LT	N-terminal **RPPDEI**	+	nd	nd	(Kellner et al., 2019)
ArtAB	N-terminal **RPPDVI**	+	nd	nd	(Kellner et al., 2019)
CARD-T	N-terminal **RSPEEI**	nd	nd	nd	–
PltA (TT)	N-terminal **TPPDVI**	nd	nd	nd	–
Other toxins					
ricin	none	–	+	–	(Spooner et al., 2008; Kellner et al., 2019)
Shiga toxin 1	none	–	–	–	(Taylor et al., 2015; Kellner et al., 2019)
Shiga toxin 2	none	–	–	–	(Taylor et al., 2015; Kellner et al., 2019)

Most ER-translocating ADPRTs have an RPPDEI-like motif within the N-terminal 20 amino acids of the A chain, although the motif in ETA begins 4 residues away from the C-terminus of its processed A chain. If ETA leaves the ER in a C- to N-terminal direction, its Hsp90 binding motif would enter the cytosol in the same RPPKGP orientation of the N-terminal toxin sequences. Hsp90 has been shown to bind peptide sequences corresponding to the N- or C-termini of toxin A chains with the RPPDEI-like motif. ER-translocating toxins that are not ADPRTs lack an RPPDEI-like motif, and the N-terminal sequences of their A chains are not recognized by Hsp90. Toxins with the motif exhibit a pattern of Hsp90-dependent intoxication and translocation. It remains to be seen if Hsp90 is required for the cellular activities of community-acquired respiratory distress syndrome toxin (CARD-T) and the pertussis-like toxin A (PltA) subunit from typhoid toxin (TT), two ER-translocating ADPRTs (Fowler et al., 2017; Ramasamy et al., 2018) that do not retain the core RPP residues of the Hsp90 binding motif. LT, Escherichia coli heat-labile toxin; ArtAB, Salmonella enterica serovar Typhimurium ADPRT; nd, not determined.

### Unique Chaperone Interactions Drive the Translocation and Cytosolic Activity of CTA1

**Figure 4:**
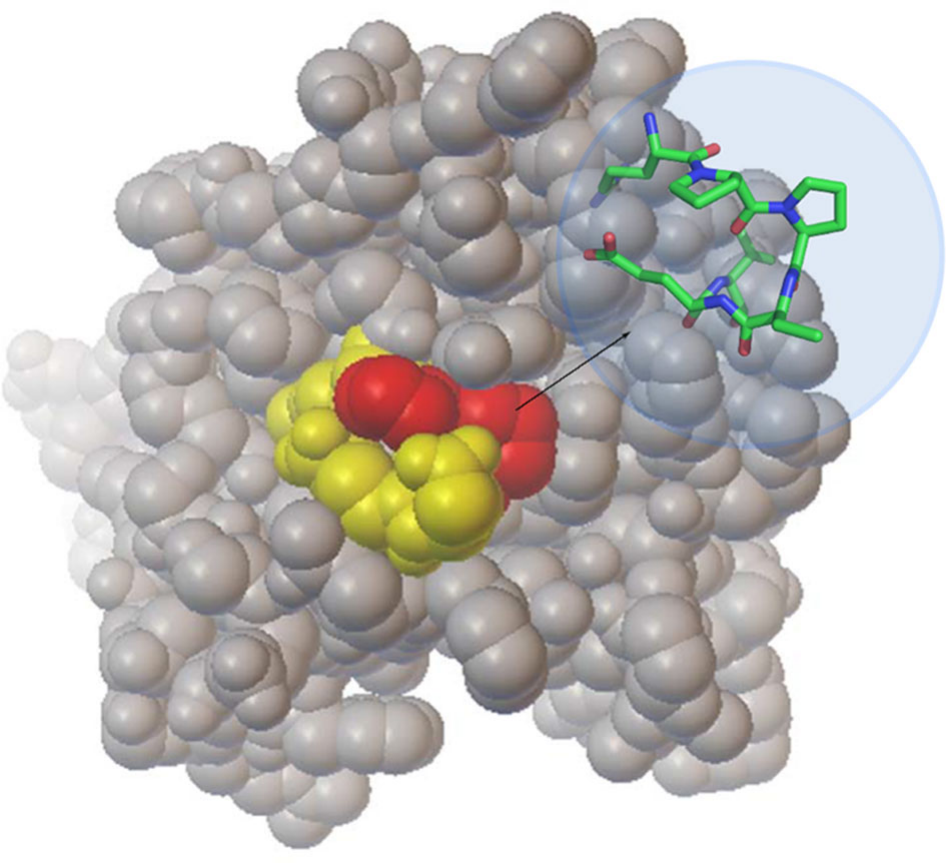
Location of the RPPDEI motif in CTA1. A space-filling model of folded, holotoxin-associated CTA1 (PDB entry 1S5E) highlights the PP residues in red and the RDEI residues in yellow. The inset stick structure provides a more detailed view of the *trans* conformations present at the PP residues of ordered CTA1.

The interplay between Hsp90 and CTA1 is highly unusual. Hsp90 recognizes a defined RPPDEI sequence in CTA1 and binds directly to this motif without the need for co-chaperones. However, it only binds to unfolded CTA1 (Taylor et al., 2010) despite the surface accessibility of the RPPDEI motif in folded CTA1 (**Figure 4**). The Hsp90-driven refolding of a client protein often results in the release of Hsp90 (Pearl and Prodromou, 2006; Schopf et al., 2017), yet Hsp90 remains tightly associated with refolded CTA1 (Burress et al., 2014). These interactions are essential for the intoxication process (Taylor et al., 2010). As detailed below, we propose proline isomerization in the RPPDEI motif is responsible for these atypical chaperone-substrate interactions.

## Hypothesis

### Proline Isomerization Plays a Key Role in Toxin Recognition by Hsp90

**Figure 5:**
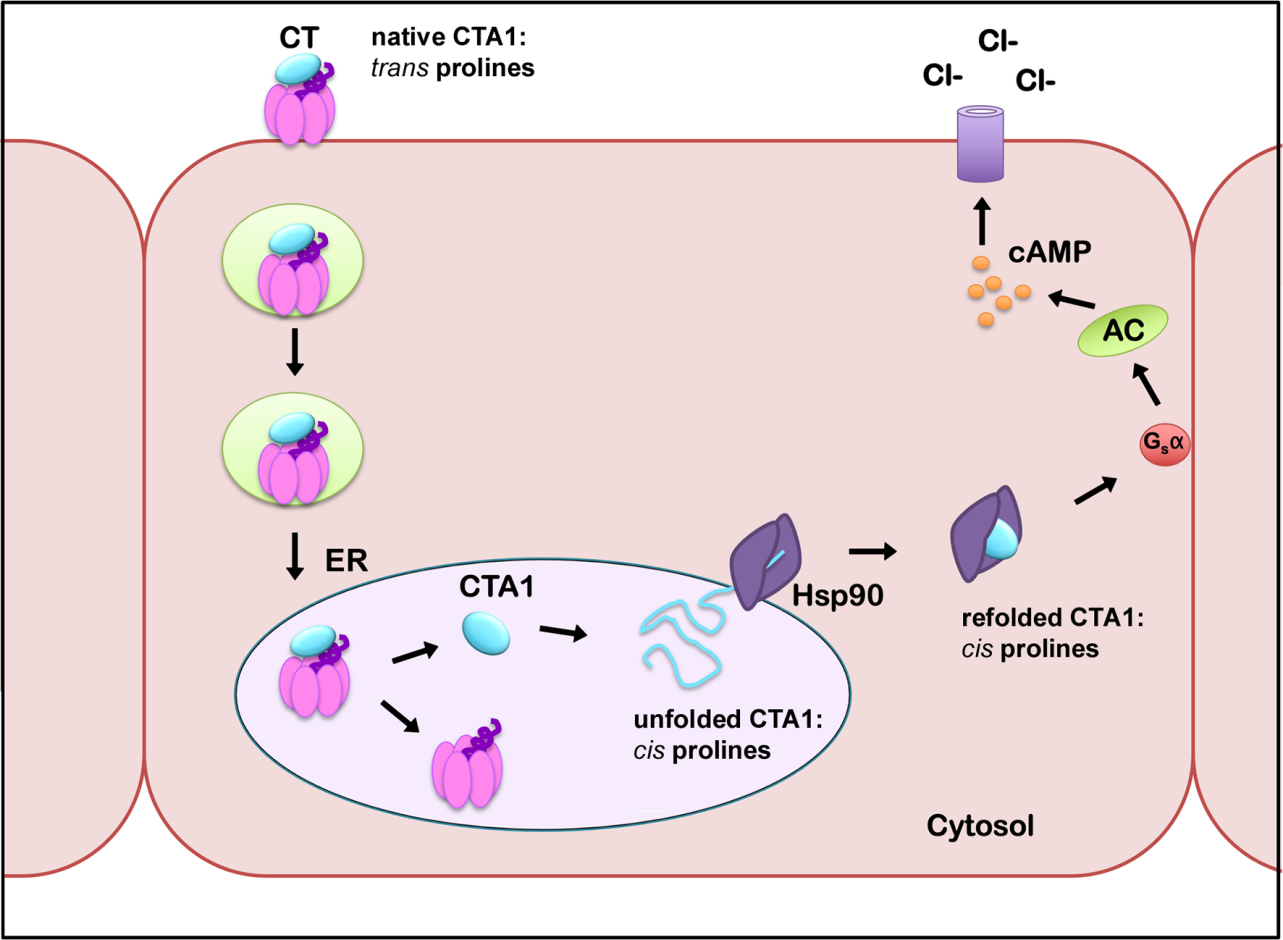
A model for the impact of proline *cis*/*trans* isomerization on CTA1-Hsp90 interactions. CTA1 enters the ER as a folded component of intact CT, with *trans* prolines in the RPPDEI motif. Separation of CTA1 from the rest of the toxin leads to its spontaneous unfolding and a hypothesized shift in its RPPDEI proline residues to the *cis* conformation. Those *cis* prolines are recognized by Hsp90 at the cytosolic face of the ER membrane after ERAD initiates the process of toxin translocation. Hsp90 then couples CTA1 refolding with CTA1 extraction from the ER translocon pore, but we predict refolding does not convert the RPPDEI prolines back to the *trans* state. Hsp90 thus remains in a stable complex with refolded CTA1 and its *cis* proline residues. Continued association of Hsp90 with CTA1 allows the otherwise unstable toxin to maintain its enzymatic activity in the host cytosol. AC, adenylate cyclase.

We predict the order-disorder-order transitions of CTA1 affect proline isomerization in its RPPDEI motif. We further predict that Hsp90 only recognizes *cis* prolines in the RPPDEI motif. When associated with the holotoxin (PDB entries 1S5E and 1XTC) (Zhang et al., 1995; O’Neal et al., 2004), CTA1 is in an ordered conformation with *trans* prolines in the RPPDEI motif (**Figure 4**, also see Materials and Methods). Thus, Hsp90 does not bind to the folded conformation of CTA1 that contains surface-exposed *trans* prolines in the RPPDEI sequence. Proline isomerization often accompanies protein unfolding (Wedemeyer et al., 2002), with Pro-Pro bonds exhibiting a greater tendency to the *cis* conformation than other Xaa-Pro bonds (Stewart et al., 1990; Pal and Chakrabarti, 1999). We accordingly predict that the ER-localized unfolding of dissociated CTA1 relaxes the structural constraints on its RPPDEI prolines and allows isomerization to the *cis* state. Hsp90 can then bind to the RPPDEI *cis* proline(s) as linearized CTA1 emerges from the ER translocon pore, thereby initiating the extraction process. If CTA1 retains its RPPDEI *cis* proline(s) after chaperone-induced refolding, this would result in the long-term association of Hsp90 with cytosolic CTA1 (**Figure 5**). Although our model is derived from studies with CTA1, it likely applies to other ER-translocating ADPRTs with an RPPDEI motif.

Proline isomerization provides a possible molecular basis for the atypical interactions between Hsp90 and CTA1. It also explains why, in contrast to endosome-translocating ADPRTs, CT does not require the action of PPIs (Burress et al., 2019): one or both of the prolines in the RPPDEI motif of disordered CTA1 is already in the proper *cis* conformation for Hsp90 recognition, whereas endosome-translocating ADPRTs rely upon PPIs to place a proline in the *cis* form required for Hsp90 binding. The presence of PPIs in the Hsp90 chaperone machinery (Tai et al., 1992; Zuehlke and Johnson, 2010; Cano et al., 2013; Li and Buchner, 2013) may likewise place the prolines of endogenous client proteins in the proper *cis* configuration for processing by Hsp90. PPI activity is currently thought to directly promote the folding of endogenous proteins and toxin A chains by shortening the rate-limiting step of proline isomerization during protein renaturation (Ernst et al., 2017), but our model suggests an additional role for PPIs in A chain translocation from the endosomes to the cytosol and, possibly, Hsp90-directed protein refolding in general. It should be noted that Hsp90 can bind directly to the A chains from several endosome-translocating ADPRTs, although this interaction has been demonstrated using an *in vitro* dot blot assay with toxins that may have contained a minor pool of protein with spontaneous conversion of prolines to the *cis* state (Dmochewitz et al., 2011; Kaiser et al., 2011; Schuster et al., 2017).

## Discussion

Proline isomerization has an established role in regulating protein function but has not yet been shown to influence substrate recognition by Hsp90. In fact, Hsp90 is not thought to recognize defined amino acid sequences (Li et al., 2012; Karagoz and Rudiger, 2015; Schopf et al., 2017). Our work with the RPPDEI binding motif for Hsp90 has demonstrated, for the first time, that Hsp90 recognizes a defined amino acid sequence in addition to the general motif of surface-exposed hydrophobic amino acid residues (Kellner et al., 2019). We posit *trans*-to-*cis* isomerization in one or both of the RPPDEI prolines accompanies the ER-localized unfolding of the toxin A chain and serves as the signal for Hsp90 binding. Endosome-translocating ADPRTs that lack the RPPDEI motif do not undergo spontaneous *trans*-to-*cis* proline isomerization during the translocation process and must therefore utilize host PPIs to prepare the toxin A chain for Hsp90 binding. Structural analysis of folded and unfolded variants of the toxin A chain by NMR (Alderson et al., 2018) or other techniques could directly test this model. The propensity for *cis* proline isomerization in the toxin A chain thus provides a new context to understand the general roles of Hsp90 and PPIs in AB toxin translocation.

## Materials and Methods

### ETA Intoxication Assay

CHO-K1 cells (American Type Culture Collection, Manassas, VA) seeded to 24-well plates were grown overnight to ~80% confluency. The cells were then exposed to 10 μM CsA (Sigma-Aldrich, St. Louis, MO) for 30 min before challenge with various concentrations of ETA in the continued presence of CsA. A second set of cells were challenged with ETA in the absence of CsA. After a 4 h incubation with toxin, all cells were washed with phosphate-buffered saline (PBS) and incubated in methionine-free medium for 30 min before supplementation of the medium with 10 μCi/mL of [^35^S]methionine (PerkinElmer, Waltham, MA) for another 30 min. The cells were subsequently bathed in ice-cold PBS containing 10% trichloroacetic acid for sequential 30 and 10 min incubations. Cell lysates generated with 0.2 N NaOH were placed in vials filled with scintillation fluid, and the acid-precipitated radiolabel that had been incorporated into newly synthesized proteins was detected with a Beckman Coulter (Brea, CA) LS6500 scintillation counter. Results from toxin-treated cells were expressed as percentages of the value obtained from a corresponding set of cells incubated without toxin. All measurements were performed in triplicate.

### PTS1 Translocation Assay

As previously described (Kellner et al., 2019), CHO cells grown to ~70% confluency in 6-well plates were transfected with 1 μg of empty pcDNA3.1 vector or the ssPTS1/pCMV/*myc*/ER expression vector (Castro et al., 2001). One set of cells was incubated overnight with 10 μM CsA. At 24 h post-transfection, all cells were lifted using PBS with 0.5 mM EDTA. Three wells of cells were pooled for each condition and centrifuged at 5,200 x g for 2 min at 4°C. After a 10 min incubation at 4°C, the cell pellet was resuspended in HCN buffer (50 mM Hepes pH 7.5, 2 mM CaCl_2_, 150 mM NaCl, 10 mM N-ethylmaleimide, protease inhibitor cocktail) containing 0.04% digitonin. After an additional 10 min at 4°C, separate membrane and cytosolic fractions were generated by centrifugation at 14,000 x g for 10 min at 4°C. The supernatant was lyophilized before resuspension in 2x sample buffer, while the pellet was directly resuspended in 2x sample buffer. Both fractions were resolved by SDS-PAGE with 15% polyacrylamide gels, using five-fold more supernatant sample than pellet. Antibody dilutions for Western blot analysis were 1:30,000 for the rabbit antibody against PT (Abcam, Cambridge, UK), 1:20,000 for rabbit antibodies against GRP94 (Stressgen, Victoria, British Columbia) or cyclophilin A (Abcam), and 1:20,000 for the secondary horseradish peroxidase-conjugated goat anti-rabbit IgG antibody (Jackson ImmunoResearch, West Grove, PA). Incubations with the primary antibodies were overnight at 4°C, while incubations with the secondary antibody were 1 h at room temperature.

### Cyclophilin Binding Assay

Two-fold serial dilutions of cyclophilin A or cyclophilin B (both from Sigma-Aldrich) at starting concentrations of 5 µg/mL were absorbed in 200 µL volumes to nitrocellulose using the BioRad (Hercules, CA) slot blot apparatus. After Ponceau stain, the membrane was blocked for 30 min at room temperature in a 4% milk solution of Tris-buffered saline with 0.05% Tween-20 (TBST). The membrane was then incubated overnight at 4°C in 15 mL of TBST/4% milk containing 5 µg of PTS1 (Aviva Systems Biology, San Diego, CA) labeled with horseradish peroxidase *via* the Lightning Link conjugation kit (Abcam). Chemiluminescence was used to visualize membrane-bound PTS1.

### Determination of Proline Isomerization State in Holotoxin-Associated CTA1

Proline conformation in the RPPDEI sequence of holotoxin-associated CTA1 can be assessed by extracting coordinates of only the atoms from the six-amino acid moiety. This subset of atoms is then saved as a text file having a .xyz extension and opened in a molecular viewer (e.g., Jmol). The measurement tool provides a direct method for measuring the relevant torsion angle. In this case, however, a visual assessment is usually sufficient as the *cis* and *trans* proline structures differ significantly.

## Data Availability Statement

The raw data supporting the conclusions of this article will be made available by the authors, without undue reservation.

## Author Contributions

AK, PC, and KT performed experiments. AK, JH, and KT conceptualized the hypothesis. AK, PC, JH, and KT analyzed the data. KT wrote the paper. AK and JH edited the paper. All authors contributed to the article and approved the submitted version.

## Funding

Research in the Teter lab on Hsp90-toxin interactions was funded by the National Institute of Allergy and Infectious Diseases of the National Institutes of Health under Award Number R01AI099493. The content is solely the responsibility of the authors and does not necessarily represent the official views of the National Institutes of Health.

## Conflict of Interest

The authors declare that the research was conducted in the absence of any commercial or financial relationships that could be construed as a potential conflict of interest.

## Publisher’s Note

All claims expressed in this article are solely those of the authors and do not necessarily represent those of their affiliated organizations, or those of the publisher, the editors and the reviewers. Any product that may be evaluated in this article, or claim that may be made by its manufacturer, is not guaranteed or endorsed by the publisher.
